# The Role of ^18^F-FDG PET/CT in Large-Vessel Vasculitis: Appropriateness of Current Classification Criteria?

**DOI:** 10.1155/2014/687608

**Published:** 2014-08-14

**Authors:** H. Balink, R. J. Bennink, B. L. F. van Eck-Smit, H. J. Verberne

**Affiliations:** ^1^Department of Nuclear Medicine, Medical Center Leeuwarden, P.O. Box 850, 8901 BR Leeuwarden, The Netherlands; ^2^Department of Nuclear Medicine, Academic Medical Center, 1105 AZ Amsterdam, The Netherlands

## Abstract

Patients with clinical suspicion of large-vessel vasculitis (LVV) may present with nonspecific signs and symptoms and increased inflammatory parameters and may remain without diagnosis after routine diagnostic procedures. Both the nonspecificity of the radiopharmaceutical ^18^F-FDG and the synergy of integrating functional and anatomical images with PET/CT offer substantial benefit in the diagnostic work-up of patients with clinical suspicion for LVV. A negative temporal artery biopsy, an ultrasonography without an arterial halo, or a MRI without aortic wall thickening or oedema do not exclude the presence of LVV and should therefore not exclude the use of ^18^F-FDG PET/CT when LVV is clinically suspected. This overview further discusses the notion that there is substantial underdiagnosis of LVV. Late diagnosis of LVV may lead to surgery or angioplasty in occlusive forms and is often accompanied by serious aortic complications and a fatal outcome. In contrast to the American College of Rheumatology 1990 criteria for vasculitis, based on late LVV effects like arterial stenosis and/or occlusion, ^18^F-FDG PET/CT sheds new light on the classification of giant cell arteritis (GCA) and Takayasu arteritis (TA). The combination of these observations makes the role of ^18^F-FDG PET/CT in the assessment of patients suspected for having LVV promising.

## 1. Introduction 

This paper focuses on the role of ^18^F-FDG PET/CT in patients with symptoms possibly related with large vessel vasculitis (LVV) and the pathophysiologically associated polymyalgia rheumatica (PMR). Patients with clinical suspicion of LVV may present with nonspecific signs and symptoms like fatigue, malaise, weight loss, anorexia, subfebrile temperatures or night sweats, and increased C-reactive protein (CRP) levels or erythrocyte sedimentation rate (ESR). This patient population may remain without a diagnosis after routine diagnostic procedures. Both the nonspecificity of the radiopharmaceutical ^18^F-FDG and the synergy of integrating functional and anatomical images with hybrid PET/CT may offer substantial benefit in the diagnostic work-up of patients with clinical suspicion for LVV. An important feature of ^18^F-FDG PET imaging is the ability to reveal increased metabolism and functional alterations that precede the morphological changes. In addition this paper discusses whether the specific characteristics of ^18^F-FDG PET/CT may shed new light on the American College of Rheumatology (ACR) classification of LVV in giant cell arteritis (GCA) and Takayasu arteritis (TA).

## 2. Background

Vasculitides are a heterogeneous group of syndromes; the 1990 American College of Rheumatology (ACR) established criteria designed to differentiate among patients with 7 types of vasculitis [1]. Historically, TA and GCA have been considered distinct diseases based on differences in age at onset, ethnic distribution, and clinical features, including predilection for involvement of certain arterial territories [2, 3].

The goals of the first International Chapel Hill Consensus Conference on the Nomenclature of Systemic Vasculitides (CHCC1994) were to reach consensus on names for the most common forms of vasculitis and furthermore to construct a specific definition for each form [4]. The CHCC1994 classification organized vasculitis according to vessel size:

- large vessels: giant cell arteritis (GCA), Takayasu arteritis (TA);
- medium vessels: periarteritis nodosa, Kawasaki's arteritis, primary CNS vasculitis, and Buerger's disease (thromboangiitis obliterans);
- small vessels: Wegener's disease, Churg-Strauss syndrome, microscopic polyangiitis, Henoch-Schonlein purpura, and essential cryoglobulinaemic vasculitis.

Because of advances in understanding the pathophysiology of vasculitis, another International Chapel Hill Consensus Conference (CHCC2012) was convened. With respect to LVV, changes in definition were made. Criteria for TA based on late effects of arterial lumen narrowing or occlusion were removed: “claudication of an extremity, decreased brachial artery pulse, difference in systolic blood pressure between arms, a bruit over the subclavian arteries or the aorta, arteriographic evidence of narrowing or occlusion of the entire aorta.” Furthermore, the previous existing gap in age between the onset of <40 years for TA of age and ≥50 years of age for GCA was closed. The following definitions were formulated.

- *Giant cell arteritis*: arteritis, often granulomatous, usually affecting the aorta and/or its major branches, with a predilection for the branches of the carotid and vertebral arteries. Often involving the temporal artery. Onset usually in patients older than 50 years and often associated with polymyalgia rheumatica.
- *Takayasu arteritis*: arteritis, often granulomatous, predominantly affecting the aorta and/or its major branches. Onset usually in patients younger than 50 years.

The term “temporal arteritis” was not regarded as a suitable alternative for GCA because not all patients have temporal artery involvement, and other categories of vasculitis can affect the temporal arteries. The CHCC2012 made notation that in patients with LVV large arteries may not be the predominant type of vessel affected because especially medium size arteries may be affected as well, or even smaller arteries, for example, ciliary and retinal arteries [5, 6]. It was recognized that the histopathological features of Takayasu arteritis and giant cell arteritis are indistinguishable, but the CHCC2012 participants did not seek to resolve the important question whether or not CGA and TA are the same disease [5].

## 3. Giant Cell Arteritis and Takayasu Arteritis

The idea that GCA and TA are part of a spectrum of conditions of a single disease was first proposed by Hall in 1973, who suggested that polymyalgia rheumatica (PMR), GCA, and TA constitute an “unholy trinity” of a single disease [7].

GCA is characterised by arterial injuries affecting the smooth muscle cells located in the media with fragmentation of the internal elastic lamina and also lymphocyte-monocyte transmural infiltration with the presence of macrophages [8]. GCA is associated with polymyalgia rheumatica (PMR) in approximately 40% of patients [9, 10]. GCA involvement of the aorta and/or its major branches may be asymptomatic or induce non-specific clinical complaints, which explains why it is often overlooked. This is underlined by the fact that late effects/complications of extracranial GCA may only be discovered after life-threatening events such as stroke, myocardial infarction, ruptured aortic aneurysm, or aortic dissections [11–14].

TA is a pan-arteritis with mononuclear infiltrates and giant cells, mostly located in the adventitia and the media [15]. TA has an estimated incidence of only 2 cases per 1 million persons. The mean age at onset is 35 years, and prevalence of the disease in women is 2–25 times higher than that in men. During the course of the disease, stenoses, occlusions, and aneurysms may occur [16]. TA is reported to be potentially life-threating, reflected in mortality rates as high as 35% at 5 years after diagnosis, similar to that seen in malignancies [17].

The pathogenesis of both GCA and TA is unknown. Both are thought to be antigen-driven cell-mediated autoimmune processes, although the specific antigenic stimulus and or stimuli have not been identified [18]. Interleukin-6 (IL-6) may be a key mediator in GCA, TA, and PMR. Patients with GCA, TA, and PMR have elevated concentrations of IL-6 in both their peripheral circulation and their inflamed tissues, and serum levels of IL-6 correlate with disease activity. IL-6 receptor blockade with tocilizumab led to clinical and serologic improvement in patients with refractory or relapsing GCA, TA, and PMR [19].

Recent observations have shown that the histopathology of arterial lesions in GCA and TA is difficult to distinguish [18, 20]. On angiography strong similarities and subtle differences in these lesions were observed between GCA and TA [21].

## 4. Temporal Artery Biopsy 

Temporal artery biopsy is considered the cornerstone of the diagnosis of GCA, which explains why review articles were published under the title “Large-Vessel Vasculitis” but dealt almost entirely with the problems of interpreting laboratory results and the results of ultrasonography and temporal artery biopsy [22]. Temporal artery biopsyis invasive and can be false negative, due to, for example, skip lesions, in 15–70% of the cases, which may considerably delay the diagnosis [23].

Large-vessel arteritis may occur in isolation, without classical features such as headache and scalp tenderness, making a clinical diagnosis difficult. In a recent study of 74 patients with subclavian/axillary GCA diagnosed by angiography and 74 control patients with temporal artery biopsy-proven GCA and without large-vessel involvement at angiography were matched for the date of first diagnosis. PMR occurred with similar frequency in both patient groups and temporal artery biopsy findings were negative in 42% of patients with large-vessel GCA. Large-vessel GCA was associated with higher concentrations of interleukin-2 gene transcripts in arterial tissue and overrepresentation of the HLA-DRB1*0404 allele, indicating differences in pathogenetic mechanisms. GCA is apparently not a single entity but may comprise several variants of the same disease. In the spectrum of clinical manifestations it often occurs without involvement of the cranial arteries [24].

This interpretation is supported by the variable phenotypes in patients at different ages that are reported in analyses of other autoimmune diseases, including systemic lupus erythematosus, rheumatoid arthritis, systemic sclerosis, and dermatomyositis [20].

## 5. Underestimation of the Prevalence/Incidence of LVV

The incidence of LVV generally mentioned in the literature is 20–30/100.000 persons (0.02%) [26]. Based on the largest retrospective series, the prevalence of involvement of extracranial vessels in GCA is around 15% [27]. The notion that there is substantial underdiagnosis of LVV is supported by several autopsy observations.

A small study from 1968 of six autopsy cases revealed involvement of the aorta and other arteries in patients with coexisting giant cell arteritis, as well in patients with PMR in whom a temporal artery biopsy was negative or clinical signs of vasculitis were absent [28]. Most convincing is a retrospective study (from 1973!) of arterial changes in 20,591 autopsy subjects in Sweden, which revealed that PMR with signs of aortic involvement is far more common than is diagnosed clinically; arteritis was found in 0.4% and only half of them had temporal arteritis [29]. The often asymptomatic course of LVV was demonstrated by a retrospective review of 1,204 aortic surgical specimens that were gathered over a period of 20 years; 52 (4.3%) were clinically and pathologically classified as idiopathic aortitis. In 31%, aortitis was associated with a remote patient history of vasculitis and a variety of other systemic disorders [30].

The heterogeneity of LVV was substantiated in a report on 72 cases of, during life, documented GCA with aortic and extracranial large vessel involvement. The disease process affected the entire aorta in 35 of 72 cases, the head and neck or upper limb arteries in 24/72 cases, and the lower limb arteries in 13/72 cases [31].

## 6. Importance of Early Diagnosis of LVV

Additionally, a late diagnosis of LVV leading to surgery or angioplasty in occlusive forms (with higher frequency in patients classified as TA) is often accompanied by serious aortic complications and a fatal outcome [32]. Manifestations are very polymorphous, with presentations that range from asymptomatic to neurologic complications. LVV has also been reported to manifest as isolated involvement of renal arteries (for which renal revascularization was required), or pulmonary arteries resulting in occlusion, and of coronary arteries, requiring bypass surgery [33–37]. Particularly in older patients visual loss in one eye was reported to be prevalent in 16–18% of patients at initial diagnosis [38, 39]. In Great Britain visual loss in patients diagnosed with temporal arteritis occurs in as much as 20% of patients [40].

Abdominal aortic aneurysms (AAA) are a substantial burden on health care. Recent studies may bridge a gap between the clinical signs and diagnosis of AAA and immune-mediated large-vessel vasculitis. Serum levels of IL-1*β*, TNF-*α*, and IL-6 were proven to be elevated in AAA patients. In AAA tissue samples, levels of TNF-*α* were found to be higher in small-sized AAAs than in large-sized AAAs and may be related to infiltration of macrophages [41, 42]. In a follow-up of 96 GCA patients that all fulfilled the ACR criteria, and of which 88 had artery biopsy specimens showing GCA (87 temporal, 1 occipital), it was found that these patients were 17 times more likely to develop a thoracic aneurysm and 2.4 times more likely to develop an AAA compared with the general population [27]. In the same cohort of 96 patients (diagnosed between January 1950 and December 1999) the median time from diagnosis of GCA to detection for AAA was 6.3 years and for thoracic aortic aneurysms 10.9 years [43].

## 7. Utility of ^**18**^F-FDG PET/CT in the Diagnosis of LVV, PMR, and Temporal Arteritis

### 7.1. LVV

In a review from 2003 and later in 2009 it was stated that in patients presenting with a prolonged inflammatory syndrome with nonconclusive signs and symptoms, the presence of diffuse increased ^18^F-FDG uptake in the wall of the aorta and its main branches may efficiently guide to the diagnosis of LVV [44, 45].

To further substantiate this statement, ^18^F-FDG PET/CT was recently performed in a series of 140 patients with inflammation of unknown origin (IUO). IUO was defined as repeated CRP values more than 20 mg/L or ESR more than 20 mm/h, with nonspecific signs and complaints, body temperature below 38.3°C (100.9°F), and without a diagnosis after conventional diagnostic procedures. The final diagnosis was related to infection in 35 patients, malignancy in 18 patients, noninfectious inflammatory disease (NIID) in 44 patients, and a variety of uncommon conditions in 7 patients. NIID was the main category with PMR in 18 patients as the first main diagnosis and LVV in 12 patients as the second most established diagnosis. Signs of PMR were seen in 3 patients with LVV, and vice versa LVV signs on the PET images in 4 patients with PMR. None of the 12 patients with LVV had clinical signs or symptoms of temporal arteritis; nevertheless biopsy was positive for GCA in 1 patient and another patient with LVV had wall thickening with ultrasonography of the temporal artery [25]. Another recent study on IUO included 88 patients aged 50 years or older with nonspecific complaints and an ESR of more than 50 mm/h for which routine evaluation revealed no diagnosis. Of the 88 included patients 18 were diagnosed with LVV and 6 with PMR, with only one of these patients eventually diagnosed with temporal arteritis [46]. In both IUO studies parameters like the proportion of patients with disease, the contribution of ^18^F-FDG PET/CT to the diagnosis, and the distribution of diseases in infection, NIID, and malignancy were similar to “fever of unknown origin” (FUO) patient populations [47–52].

**Figure 1:**
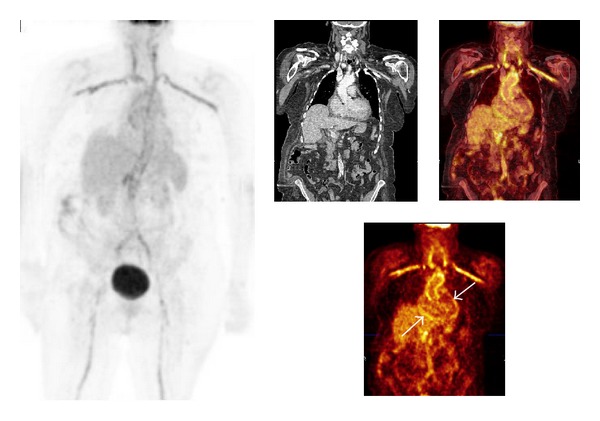
Patient history: lack of appetite and pain between the shoulder blades. Cardiologic evaluation and gastroscopy negative. CRP 224 mg/L. Increased ^18^F-FDG uptake in the aorta and its main branches and less intense FDG uptake in the distal abdominal aortic wall; corresponding CT slices show calcifications here. The mild increased perisynovial ^18^F-FDG uptake at both shoulders, which might be indicative of associated PMR. The increased ^18^F-FDG uptake at the pericardium is suggestive of pericarditis (white arrows). Note: patient had a carbohydrate restricted diet for 2 days before the ^18^F-FDG PET/CT investigation to decrease the ^18^F-FDG uptake in the myocardium. Patient had a TIA one year earlier and subsequent carotid artery desobstruction. LVV was not suspected at that time; no immunosuppressive therapy was given. After the diagnosis of LVV patient was in remission during 4 years with Prednisolon orally tapered from 10 to 7.5 and later 5 mg daily. Due to relapse Prednisolon was increased to 15 mg daily. Patient died 5 years after the diagnosis of LVV after a severe CVA.

In the literature reports on LVV—with the presence of diffuse and mildly intense ^18^F-FDG uptake in the wall of the aorta and its main branches—are numerous and mostly comprise large patient numbers (Figure 1). The reported initial response to immunosuppressive therapy is better in LVV patients with nonspecific symptoms at the time of diagnosis, compared to patients that comply to the ACR 1990 criteria with measurable effects of arterial stenosis [49, 53–61].

Reports on patients with LVV limited to the aortic arch or only in isolated arteries; for example, the carotid or vertebral arteries are scarce (only case reports) and the images display a more intense ^18^F-FDG in the aortic or arterial wall, compared to patients with LVV in the entire wall of the aorta and its main branches. A relatively high number of patients have symptoms at the time of diagnosis due to arterial occlusion and both relapse and progression (metabolic and angiographic) despite immunosuppressive therapy are reported [34–37, 62–64].

### 7.2. Polymyalgia Rheumatica

**Figure 2:**
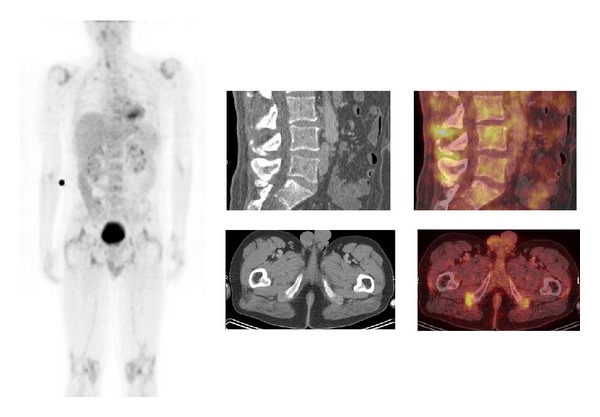
A 48-year-old man presented with initial painful calves followed by progressive painful arms and legs, shoulders, and knees. No hydrops or other clinical signs of arthritis. Normal body temperatures; CRP level, 84 mg/L; ESR, 41 mm/h; normal routine laboratory values; rheumatoid factor negative; cyclic citrullinated peptide antibody test negative; serum angiotensin-converting enzyme, 10.3 units/L; antinuclear antibody test negative; and anticytoplasmic autoantibodies negative. Urine sediment: albumin trace. Glomerular basal membrane antibody test negative. Viral serology negative. Chest X-ray and abdominal ultrasonography without abnormalities. X-ray of hands, feet, and knees revealed no erosive changes. Ultrasonography of the hips revealed no abnormalities. Also ^18^F-FDG PET/CT showed pathological perisynovial uptake at the major joints, as well as pathological lumbar interspinous uptake in the soft tissue (bursae) lateral to both of the greater trochanters and dorsal to both of the tuber ischii. The diagnosis of PMR was made; after treatment with steroids, the patient became pain free, and the CRP values remained less than 10 mg/L [25].

^18^F-FDG PET/CT images of patients with PMR reveal a characteristic pattern of pathologic ^18^F-FDG uptake in the soft tissue and ligaments (perisynovitis or enthesitis) around the shoulders and hips, lumbar (and in many cases cervical) spinous processes, and ischial tuberosities (Figure 2) [65, 66]. ^18^F-FDG PET/CT may show 2 different patterns of interspinous uptake: focal and diffuse. Diffuse uptake may reflect interspinous ligament inflammation; focal interspinous uptake may represent interspinous bursitis [66]. After chronic ligamentous interspinous inflammation, interspinous bursae may develop, leading to interspinous bursitis [67]. MRI is widely used to detect bone marrow edema and enthesitis in patients with spondyloarthritides (SpA). ^18^F-FDG PET/CT may provide an alternative diagnostic method and will likely contribute to the early diagnosis of SpA in PMR [68]. Both LVV and PMR may be detected, in the early onset of the disease, by ^18^F-FDG PET/CT. In some patients LVV is associated with PMR and vice versa [69, 70].

### 7.3. Temporal Arteritis

The first introduced stand-alone PET cameras provided a spatial resolution of 10 mm; a study from 2004 concluded, as a consequence, that stand-alone ^18^F-FDG PET was not yet suitable for the diagnosis of temporal arteritis and therefore could not replace invasive biopsy [71]. Ongoing improvements in technology created an evolution in spatial resolution from 6 mm to 4 mm. In recent years increased ^18^F-FDG uptake is visualized in patients with arteritis temporalis [72]. The most recently introduced PET/CT cameras claim a 2.5 mm spatial resolution for the PET component under optimal conditions. It is therefore to be expected that pathologic ^18^F-FDG uptake in temporal arteritis will be reported more frequently. However, in patient populations with prolonged inflammatory parameters and nonspecific complaints and a positive ^18^F-FDG PET/CT result for the diagnosis of LVV, temporal artery biopsy was negative in 50% [49, 73].

## 8. Specificity and Differential Diagnosis of Pathologic ^**18**^F-FDG Uptake in the Arterial Wall

Many patients, assessed for malignant disease but without a history of vasculitis, may show some uptake of ^18^F-FDG in, for example, the walls of the aorta, the subclavian arteries, and are with highest incidence in the iliofemoral arteries [74, 75]. Therefore, nuclear medicine physicians and other PET/CT practitioners have to be aware of the clinical significance of the different vascular patterns. As ^18^F-FDG accumulates in macrophage-rich areas, it cannot distinguish between sterile inflammation—such as large vessel vasculitis—and infectious inflammation. In the differential diagnosis, the pattern and the localisation of the vascular involvement as well as the intensity of ^18^F-FDG vascular uptake in the arterial wall should be taken into account for interpretation and especially differentiated from blood pool activity.

The differentiation of atherosclerosis from large vessel vasculitis is considered less problematic with PET/CT compared to a stand-alone PET [76]. Atherosclerosis usually displays a patchwork of partially normal vessel wall, focal inflammation, and calcifications. In terms of patient age, arterial inflammation precedes calcification; a study from 2005 with ^18^F-FDG PET/CT showed that inflammation and calcification only had overlap in <2% of cases, suggesting that calcification and focal arterial inflammation represent different stages in the evolution of atheroma (Figure 4) [44, 77]. Future studies will tell if this simple interpretation of the images holds true; the possible link between vasculitis, inflammation, and atherosclerosis was already suggested more than a decade ago [78–80]. Subsequent studies showed that waist circumference and atherogenic risk factors were the strongest determinants of a patchy ^18^F-FDG arterial uptake pattern, and for that reason “metabolic syndrome” was associated [74, 81].

In addition to GCA and Takayasu arteritis, other rheumatologic disorders, including rheumatoid arthritis, systemic lupus erythematosus, Wegener granulomatosis, Behçet's disease, polyarteritis nodosum, and microscopic polyangiitis, may lead to aortitis. In the case of rheumatoid associated aortitis, rheumatoid nodules are reported in the aortic wall in up to 50% of pathological specimens [82]. Furthermore, aortitis was reported in the HLA-B27-associated seronegative spondyloarthropathies, Reiter's syndrome, and ankylosing spondylitis [83]. Case reports exist on aortitis associated with sarcoidosis [84]. Cogan's syndrome is an unusual disorder characterized by episodes of interstitial keratitis and vestibuloauditory dysfunction (i.e., eye and ear symptoms); aortitis occurs in up to 10% of cases of Cogan's syndrome [62, 85]. Syphilitic aortitis, localized in only the wall of the ascending aorta, is reported in several recent case reports [86–88]. Aortitis also occurs in association with idiopathic retroperitoneal fibrosis (Ormond's disease), inflammatory abdominal aortic aneurysm, and perianeurysmal retroperitoneal fibrosis, a group of clinical disorders now categorized as chronic periaortitis [89].

However, the above-mentioned disease entities are different from LVV in that the inflammation is limited to the aorta and periaortic tissues rather than a manifestation of a widespread vasculitis of the aorta and its main branches.

As ^18^F-FDG PET/CT will be more frequently used as a screening tool in more complex diagnostic settings like fever and inflammation of unknown origin, a routine investigator-independent strategy for establishing the diagnosis of LVV is needed. In this respect semiquantification might be helpful: a ratio of the ^18^F-FDG maximal standardized uptake values (SUVmax) of the aorta-to-liver appeared more reliable compared to the SUVmax of the aorta-mediastinum ratios for the diagnosis of LVV and was not affected by minor inflammation-associated changes in hepatic metabolism [56].

## 9. Sensitivity and Specificity of ^**18**^F-FDG-PET/CT in Comparison to Other Imaging Modalities

In the knowledge that ultrasonography, MRI, arteriography, and PET/CT have proven useful image techniques in the diagnostic approach of LVV or suspicion of LVV, it remains difficult to compare the different imaging modalities. Results have to be interpreted with caution as metabolic changes in the arterial wall usually precede the anatomic changes [90–94]. Furthermore, the instigating inflammatory process may have subsided in arterial stenosis or aortic aneurysm. Problems rise therefore in the interpretation of ^18^F-FDG PET/CT results in the many reports that describe patient populations that met the ACR 1990 classification criteria for GCA (i.e., with temporal artery abnormalities and/or a positive biopsy for arteritis) and TA (i.e., with clinical and measurable effects of arterial stenosis) (Table 1). Furthermore, in the majority of studies, patients were already receiving steroids, which negatively influences the sensitivity of ^18^F-FDG PET for inflammatory processes, while sequelae like oedema and aortic/arterial wall thickening (CT or MRI) need more time to respond to therapy.

Reported discrepancies in ^18^F-FDG PET/CT results, disease activity measured by inflammatory parameters, and radiologic evaluation with MRI may very well be caused by interpreting monitoring immunosuppressive therapy response with ^18^F-FDG PET/CT as an initial staging procedure [54, 58, 64].

The active phases of recruitment, activation, migration, and infiltration of T cells, macrophages, and leucocytes usually precede the appearance of inflammatory oedema; ^18^F-FDG PET/CT may therefore be positive at an earlier stage than an MRI scan [95]. Also angiography is suboptimal for the diagnosis of LVV because it detects only the late anatomical changes such as arterial wall abnormalities, arterial stenosis, or aortic aneurysm [96]. However, the chronic inflammation in asymptomatic AAAs was not sufficiently metabolically active to result in detection with ^18^F-FDG PET/CT. Despite that histologic examination of large asymptomatic AAAs (range 52–66 mm) and small AAAs (range 34–40 mm) showed residual inflammatory cell infiltration with T cells and macrophages [97].

In addition the lack of a gold standard creates problems in the calculation of sensitivity and specificity; in some reports LVV diagnosed by ^18^F-FDG PET/CT was confirmed by histology (of the temporal artery while patients with LVV have involvement of the temporal artery in only approximately 50% of the cases!) or MRI angiography [61] (Table 2).

Or the diagnostic accuracy and sensitivity/specificity of an international expert panel was calculated and compared to computer calculated logistic regression models as a reference to assess the impact on clinical management with and without ^18^F-FDG PET results [55].

Because GCA and TA were considered as two independent distinct diseases, patients with Takayasu arteritis (i.e., patients younger than 50 years) were excluded in reports and reviews [98].

In conclusion, a negative temporal artery biopsy, an ultrasonography without an arterial halo, or an MRI without aortic wall thickening or oedema do not exclude the presence of LVV and should therefore not exclude the use of ^18^F-FDG PET/CT when LVV is clinically suspected.

## 10. Cost-Efficacy

In the “new” era of health care technology assessment (HTA), the costs of a diagnostic procedure should be weighed against its effectiveness in daily clinical practice. ^18^F-FDG PET/CT has the ability to visualize the early onset of inflammatory processes within a whole body scan; positive findings correlate well with clinical and laboratory markers of inflammation, in particular C-reactive protein. The level of ^18^F-FDG uptake may also provide prognostic information in LVV [96]. The intensity of thoracic aortic wall ^18^F-FDG uptake at the time of diagnosis correlated with later increased aortic diameter (as measured by CT) after a mean of 46.7-month follow-up (adjusted for age, sex, hypertension, diabetes, cholesterol levels, erythrocyte sedimentation rate, and CRP). On multivariate analysis, only ^18^F-FDG uptake at baseline remained significantly associated with increased thoracic aortic diameter (*P* = 0.039) [59].

Repeated ^18^F-FDG PET/CT procedures involve expenses and radiation exposure to patients with vasculitis [99]. On the other hand, in patients diagnosed with PMR and without signs of LVV, an additional ^18^F-FDG PET/CT will probably offer no advantage over the traditional follow-up of PMR patients, based on clinical evaluation and periodic determination of inflammatory laboratory parameters [100]. However, in patients diagnosed with LVV and treated with steroids, both normalization of laboratory data and symptomatic improvement of the patient correlated well with normalization of ^18^F-FDG uptake in the large-vessel walls. CT and MRI frequently show residual abnormal findings even after symptoms have completely resolved and with discrepancies concerning ESR and CRP laboratory data [101, 102].

**Figure 3:**
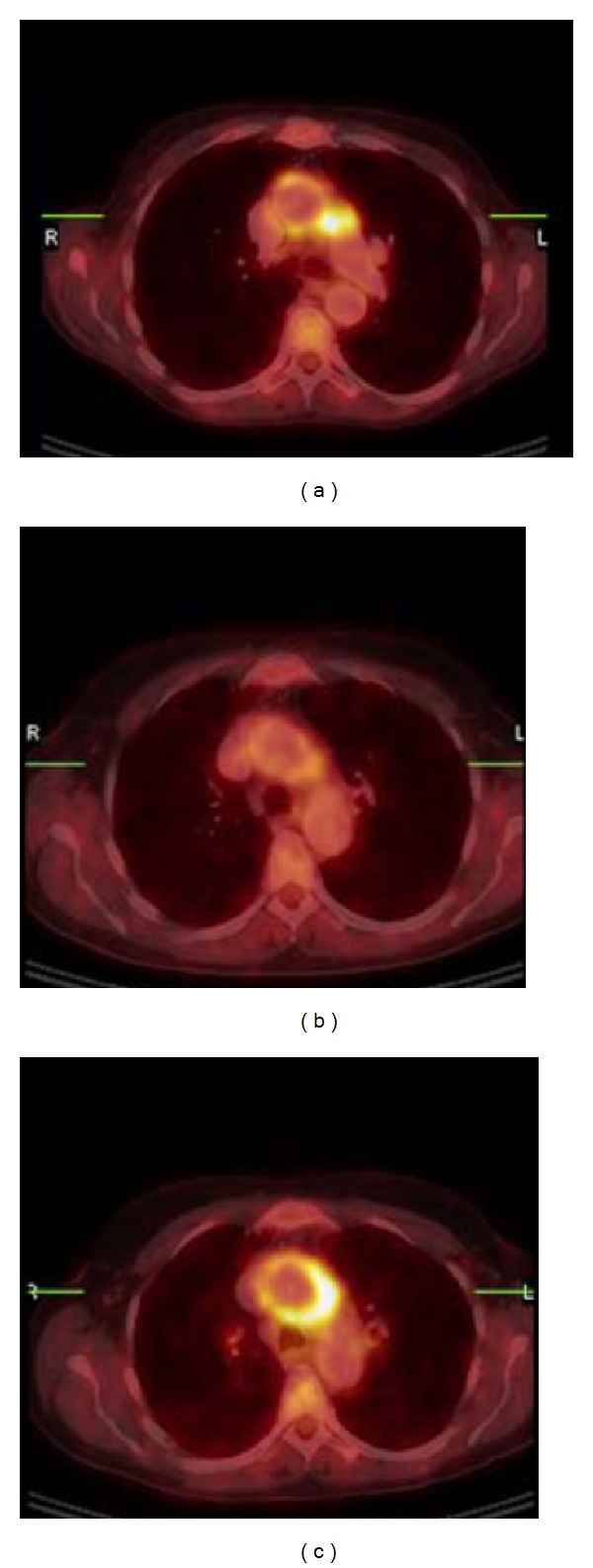
Aortitis in Cogan's syndrome. (a) Transverse hybrid PET/CT slice; pathological uptake in the wall of the aortic arch, more intense in the lateral wall and perivascular space adjacent to the truncus pulmonalis. (SUVmas 12, ESR 52 mm/h, CRP53 mg/L). (b) Follow-up PET/CT showed clearly decreased uptake in the aortic arch after 3 weeks treatment with methyl-Prednisolon i.v. and Prednisolon orally. (SUVmax 4, ESR 11 mm/h, and CRP < 2 mg/L). (c) Second follow-up PET/CT 6 months later (patient was in a stable condition with methotrexate and low-dose prednisone) with again high uptake in the wall of the aortic arch, with higher intensity in the lateral wall and perivascular space adjacent to the truncus pulmonalis. Methotrexate and prednisone were both increased to 20 mg/day (SUVmax 13, ESR 24 mm/h, and CRP 14 mg/L).

**Figure 4:**
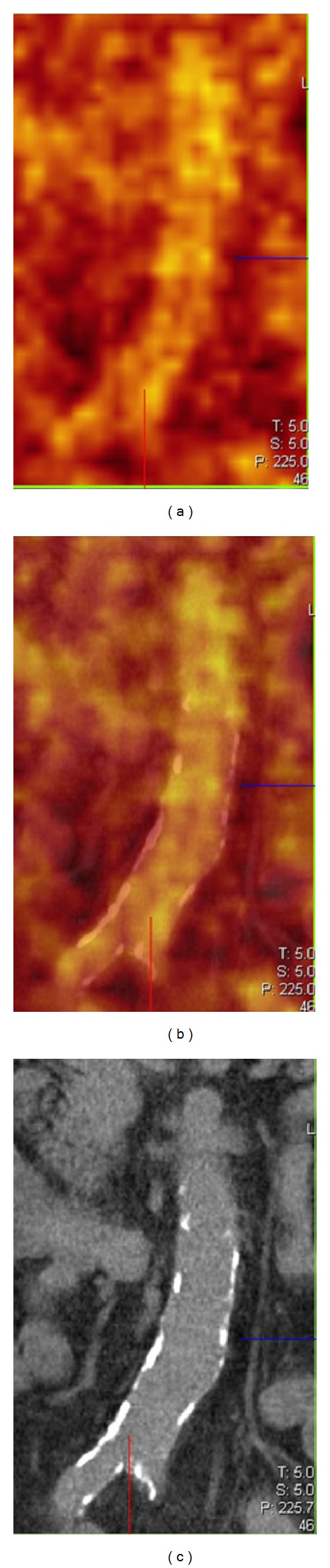
From left to right: PET, PET/CT, and CT coronal slices of atherosclerosis of the lower abdominal aorta. The focal and patchy increased FDG uptake representing inflammation and calcifications do not show overlap.

Patients diagnosed with LVV probably need to be classified in different risk groups, as already suggested in 1999 [24]. ^18^F-FDG PET/CT is able to diagnose patients with LVV without cranial or cervical artery involvement, which is clinically relevant as patients with arteritis temporalis are probably more prone to develop thoracic aortic dilatation or arterial occlusion, and therefore need a more intensive treatment and close monitoring as compared to patients with isolated LVV or PMR [100, 103]. Those patients with localized and more intense ^18^F-FDG uptake limited to only one artery or, for example, in the cervical arteries need even more close monitoring due to a higher risk of relapse and vascular complications; for example, aortitis in Cogan's syndrome is indistinguishable from TA. During the course of Cogan's syndrome aortic insufficiency may develop that may require valve replacement. Reports describe relapse and progression (both metabolic and angiographic) despite immunosuppressive therapy (Figure 3) [62, 104–106].

## 11. ACR Classification Criteria of LVV in Clinical Practices Using ^**18**^F-FDG-PET/CT

The ACR vasculitis classification criteria were never intended for diagnostic purposes, as pointed out by Hunder and colleagues [1]. Nevertheless, clinicians often use these criteria to diagnose LVV. The ACR 1990 criteria for GCA and TA were based on the diagnosis of advanced cases. The criteria for GCA were developed at a time when involvement of the aorta and its main branches was not a well-recognized feature of GCA, and instead there was a focus on the involvement of cranial arteries of the disease. Already in 1998 it was concluded that the ACR 1990 classification criteria function poorly in the diagnosis of the specific vasculitides. Patients who do not have a vasculitis syndrome may meet very well the ACR criteria, and on the other hand patients who have a specific type of vasculitis may meet criteria for more than one of the vasculitides as specified by the ACR criteria [107].

A retrospective review of 75 patients with TA and 69 patients with GCA (as defined by the ACR 1990 criteria) compared the signs and symptoms of disease. Patients with GCA had a greater prevalence of jaw claudication (GCA 33%, TA 5%), blurred vision (GCA 29%, TA 8%), diplopia (GCA 9%, TA 0%), and blindness (GCA 14%, TA 0%). The perception of clinicians that the widely recognized classic manifestations are distinct for GCA and TA may have led to bias in history taking, physical examination, and selection of diagnostic studies. This bias might have impaired the recognition of similarities between GCA and TA [20].

The strict implementation of the ACR criteria in combination with ^18^F-FDG PET/CT may create a significant source of confusion in the statistics and a significant bias in how the data are gathered in the classification of LVV. This is, for example, illustrated as patients with isolated and intense pathologic ^18^F-FDG uptake in the vertebral arteries and with neurologic symptoms were diagnosed TA in one case report and as GCA in another, given an age of more or less than 50 years at the onset of disease [34, 35]. It is also puzzling that patients with a homogeneous pattern of increased ^18^F-FDG uptake in the aorta and its main branches and no arterial wall abnormalities on the corresponding CT slices are diagnosed as either TA or GCA, with only their age (≤ or ≥ 50 years) as the discriminating parameter [54, 55, 58, 60, 98, 108, 109]. TA is reported to be potentially life-threating, reflected in high mortality rates related to arterial stenoses, occlusions, and aortic aneurysms. Notwithstanding, complicated courses of GCA (mean age 66 years) were reported, with persistent inflammatory markers, arterial stenoses, and aortic aneurysms despite immunosuppressive therapy and ^18^F-FDG PET showing signs of active vasculitis [92].

To further describe the confusion that ^18^F-FDG PET/CT results may create in LVV classification, patients older than 50 years with a homogeneous pattern of increased ^18^F-FDG uptake in the wall of the aorta and its main branches were erroneously reported as having TA instead of GCA [54, 58, 101, 110].

## 12. Conclusions and Future Perspectives

This review illustrates the usefulness of ^18^F-FDG PET/CT in the heterogeneity of the large vessel vasculitides. In patients with a clinical suspicion for LVV, ^18^F-FDG PET/CT is able to diagnose LVV especially at the early onset of disease. In contrast to the ACR 1990 criteria for vasculitis, based on late LVV effects like arterial stenosis and/or occlusion, ^18^F-FDG-PET/CT sheds new light on the classification of GCA and TA and strengthens the notion that GCA and TA are more likely to be different expressions of a common histopathological entity. ^18^F-FDG PET/CT is a powerful metabolic imaging tool that may help to improve the current classification system, based on the intensity of the ^18^F-FDG uptake and its distribution pattern:

- isolated polymyalgia rheumatica (PMR) with pathologic ^18^F-FDG uptake in the soft tissue and ligaments (perisynovitis or enthesitis) around the shoulders/hips and other major joints, lumbar/cervical spinous processes, and ischial tuberosities;
- diffuse and mildly intense ^18^F-FDG uptake in the wall of the aorta and its main branches and without arteritis temporalis, PMR, or anatomic arterial wall abnormalities;
- diffuse and mildly intense ^18^F-FDG uptake in the wall of the aorta and its main branches with arteritis temporalis, PMR, or anatomic arterial wall abnormalities;
- intense and focal ^18^F-FDG uptake in the aortic arch or intense ^18^F-FDG uptake in isolated arteries.

In our opinion it is redundant to classify patients with a positive ^18^F-FDG PET/CT for LVV in ≤50 years or ≥50 years of age.

An unintended problem that arises from the diagnosis of the early onset of LVV with ^18^F-FDG PET/CT is the longer time period patients will be exposed to the adverse effects of glucocorticoids resulting in more pressure on the major need for more specific drugs to induce and maintain remission and to reduce the cumulative adverse effects of long-term glucocorticoid exposure. However, ^18^F-FDG PET/CT may be useful in the increasing need for therapy monitoring resulting in better treatment planning and possibly reducing long-term adverse treatment effects.

## Figures and Tables

**Table 1:** Definitions for large vessel vasculitis according to the American College of Rheumatology (ACR) 1990 criteria for the classification of giant cell arteritis and Takayasu's arteritis and the definitions revised by the 2012 International Chapel Hill Consensus Conference on the Nomenclature of Vasculitides (CHCC2012).

	ACR 1990 criteria	CHCC2012 definition
Large-vessel vasculitis (LVV)		Vasculitis affecting large arteries more often than other vasculitides Large arteries are the aorta and its major branches

Giant cell arteritis (GCA)	Age at onset of disease ≥ 50 yr New headache Temporal artery abnormality Elevated erythrocyte sedimentation rate Abnormal findings on biopsy of temporal artery Diagnosis: at least 3/5 criteria	Arteritis, often granulomatous, usually affecting the aorta and/or its major branches, with a predilection for the branches of the carotid and vertebral arteries Often involves the temporal artery Onset usually in patients older than 50 years and often associated with polymyalgia rheumatica Arteritis, often granulomatous, predominantly affecting the aorta and/or its major branches

Takayasu arteritis (TA)	Age at onset of disease ≤ 40 yr Onset usually in patients younger than 50 years Claudication of an extremity Arteritis, often granulomatous, Decreased brachial artery pulse predominantly affecting the aorta and/or its major Difference in systolic blood pressure between arms branches A bruit over the subclavian arteries or the aorta Arteriographic evidence of narrowing or occlusion of the entire aorta Diagnosis: at least 3/6 criteria	Onset usually in patients younger than 50 years Arteritis, often granulomatous, predominantly affecting the aorta and/or its major branches

**Table 2:** Sensitivity and specificity of different imaging modalities for LVV with their pathognomonic/typical imaging findings. (data on US and MRI from [111]).

	Pathognomonic/typical findings	Sensitivity/specificity
(Color-doppler) ultrasonography	(i) Edema, halo around the (temporal) artery (ii) Stenosis, increased blood flow velocities (iii) Occlusion, absent colour signal	Sens. 87%, Spec. 96% (for late effects of arteritis temporalis)

MRI	(i) Wall thickening (ii) Increased mural gadolinium contrast enhancement	Sens. 81%, Spec. 97% (for late effects of LVV)

^ 18^F-FDG PET/CT	Increased ^18^F-FDG uptake in walls of aorta and main cervical and thoracic branches	(i) Able to detect early inflammation without (late) effects like oedema, wall thickening, or arterial stenosis or aortic dilatation (ii) How to calculate sensitivity and specificity in lack of a gold standard?
